# Metal Accumulation by *Jatropha curcas* L. Adult Plants Grown on Heavy Metal-Contaminated Soil

**DOI:** 10.3390/plants9040418

**Published:** 2020-03-30

**Authors:** Juan Francisco García Martín, María del Carmen González Caro, María del Carmen López Barrera, Miguel Torres García, Douglas Barbin, Paloma Álvarez Mateos

**Affiliations:** 1 Departamento de Ingeniería Química, Facultad de Química, Universidad de Sevilla, C/ Profesor García González, 1, 41012 Seville, Spain; maricarmengnz@hotmail.com (M.d.C.G.C.); mlopez91@us.es (M.d.C.L.B.);; 2 Departamento de Ingeniería Energética. E.T.S. de Ingeniería, Universidad de Sevilla, Camino de los Descubrimientos, s/n, 41092 Seville, Spain; 3 Department of Food Engineering, University of Campinas (UNICAMP), Rua Monteiro Lobato, 80, Cidade Universitária, Campinas-SP 13083-862, Brazil

**Keywords:** bioaccumulation, heavy metals, *Jatropha curcas*, mining soil, translocation factor

## Abstract

*Jatropha curcas* has the ability to phytoextract high amounts of heavy metals during its first months just after seeding. Notwithstanding, there is scarce information about metal uptake by adult *J*. *curcas* plants. To shed light on this issue, 4-year-old *J. curcas* L. plants were planted in a soil mixture of peat moss and mining soil (high metals content), and the biomass growth and metal absorption during 90 days were compared with those of plants growing in peat moss. The main metal found in the mining soil was Fe (31985 mg kg^−1^) along with high amounts of As (23717 mg kg^−1^). After the 90-day phytoremediation, the plant removed 29% of Fe and 44% of As from the soil mixture. Results revealed that *J. curcas* L. translocated high amounts of metals to its aerial parts, so that translocation factors were much higher than 1. Because of the high translocation and bioaccumulation factors obtained, *J. curcas* L. can be regarded as a hyperaccumulator plant. Despite the great capacity of *J. curcas* L. to phytoremediate heavy-metal-contaminated soils, the main drawback is the subsequent handling of the metal-contaminated biomass, although some potential applications have been recently highlighted for this biomass.

## 1. Introduction

Soil contamination caused by heavy metals such as As, Cd, Cr, Cu, Pb and Zn remains as a critical environmental problem that negatively affects ecosystems and, as a consequence, human health [1]. The mining and mineral processing of sulphide ore deposits are one of the main anthropogenic sources of heavy metals in soils [2]. Among the different procedures for in situ decontamination of these soils, phytoremediation can be highlighted, since it is eco-friendly and less expensive than the conventional physicochemical techniques for soil remediation [3].

Phytoremediation is a type of bioremediation that involves plants degrading or immobilizing contaminants in soil and groundwater. This may improve soil fertility and increase organic matter content [4,5]. There are six main types of phytoremediation: phytostabilization (phytosequestration), rhizodegradation, phytohydraulics, phytoextraction (phytoaccumulation), phytovolatilization and phytodegradation. Phytostabilization is the most suitable technique for mining soils, since it involves metal absorption by roots and immobilization inside them. Thus, plants uptake the metals without affecting topsoil, thus conserving its utility and fertility. Notwithstanding, there are plants that do not retain the metals absorbed by the roots in them but translocate these metals into the plant aerial parts. This root-to-shoot translocation is a hazard for the restoration of mining areas, since metals can pass from stems and leaves to birds and small herbivore mammals of the surroundings, and from them to carnivores and to the human chain.

*Jatropha curcas* L. is a plant belonging to the Euphorbiaceae family that can be found in tropical and subtropical regions [6,7]. This plant can survive and grow on marginal, eroded and depleted lands, and requires little water to grow, so it has been proposed for phytoremediation of metal-contaminated areas [8,9,10,11]. In a previous work, we assayed the phytoremediation of highly heavy-metal contaminated mining soils with *J. curcas* L. [11]. To do this, in that work we firstly sowed *J. curcas* L. seeds in vermiculite and, when the seeds germinated, the seedlings were acclimatized first in a climate chamber (6 weeks) and then in a greenhouse (another six weeks) simulating the climate of the South of Spain, leading to 70–90 cm tall plants. The plants were then grown in different mixtures of mining soils for 60 days to study the adaptation of *J. curcas* L. to these soils and, subsequently, the soil mixtures where the plant adapted the best were used for phytoremediation over 120 days, analysing the concentration of heavy metals in soils, roots, stems and leaves at days 0 and 120. We found that *J. curcas* L. absorbed huge amounts of Fe (> 3000 mg Fe kg^−1^ plant), which was the main metal found in the soils (initial concentration higher than 30000 mg Fe kg^−1^ soil). Besides, the initial concentration of metals within the range 10–1000 mg metal kg^−1^ soil such as Cr, Ni, Cu, Zn and Pb were reduced in that work between 30 and 70%, except As, which was barely absorbed by *J. curcas* L. Furthermore, metals with initial concentrations lower than 10 mg metal kg^−1^ soil, such as Cd, Hg and Sn, were fully removed from soils. These results were considered as promising. Nevertheless, the translocation factors of Cr, Ni, Pb and Zn were high, which is, indeed, an actual hindrance for the phytoremediation of soils containing high metal concentrations. This is in contrast with the results obtained by other authors, who stated that Cd, Cr, Hg, Pb and Zn levels absorbed by the aerial parts of *J. curcas* were relatively low, leading to translocation factors much lower than 1 [9,10].

The high translocation factors found in our previous work could be related to the growth of the plant, since the experiments were carried out using shoots of the plant. It could be assumed that the metals translocation from roots to aerial parts could be slowed down in adult plants. Therefore, the main aim of this research was to shed light on this hypothesis. Hence, we repeated the phytoremediation experiment carried out in our previous work under the most suitable conditions, but using adult plants instead of shoots of *J. curcas*. As the main result from this research, we can state that the starting hypothesis was not fulfilled, because high metal translocation factors were observed in the adult plants.

## 2. Results

### 2.1. Analysis of Soils at Days 0 and 90

The metal content of the soil taken from the heap area, with the highest concentration of leachate metals, of the Santo António mine in Penedono (Portugal) is illustrated in Table 1. Since this mine is intended for the extraction of gold from arsenopyrite, Fe and As (semimetal) were the main metals found in the soil (Table 1). Due to the extremely high metal concentration of the mine soil, and based on a previous research [11], a soil mixture (CS) containing 20% (wt.) mine soil and 80% (wt.) peat moss was selected to study the metal uptake by *J. curcas* L., and the results were compared with the metal uptake of plant grown in peat moss (NCS). Table 1 also shows the content in ten metals, quantified by ICP-OES, in NCS and CS.

Table 2 shows the percentage of reduction of metals in NCS and CS after 90-day phytoremediation. It can be observed that there was a notable reduction in all the analysed metals in both soils.

**Figure 1:**
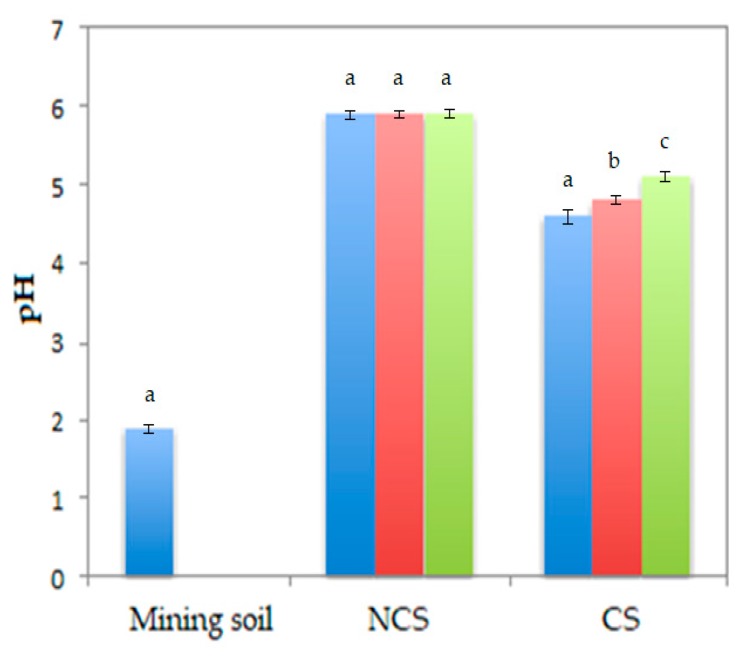
pH of the mining soil, NCS and CS at days 0 (blue bars), 30 (red bars) and 90 (green bars). Different letters in the same soil indicate significant differences in the pH value according to Duncan’s multiple range test (*p* ≤ 0.05).

The pH of the soils was measured at three given times in this study. To be specific, the day before planting (day 0), at day 30 of phytoremediation, and after the phytoremediation period (day 90), as shown in Figure 1. There were no significant differences among pH of NCS at different days. By contrast, the values of pH of CS at days 0, 30 and 90 were significantly different.

### 2.2. Analysis of the Plants Grown in NCS and CS at Days 0 and 90

The size of the plants was measured at the beginning and at the end of the 90-day phytoremediation period. The statistical analysis confirmed there were no noticeable changes in size due to the maturity of the plants, thus the height of the plant, and the diameters of the main stem and ramified stems (branches) remained constant (Table 3). After the first four years of *J. curcas* L, the plant reaches its highest size, thus the variation in size during the following years is minimum.

**Figure 2:**
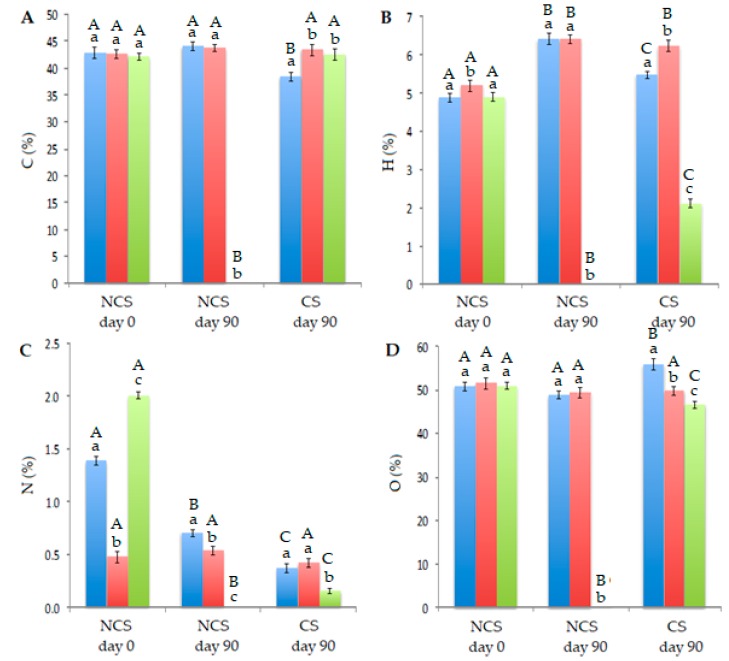
Percentages of carbon (**A**), hydrogen (**B**), nitrogen (**C**) and oxygen (**D**) in roots (blue bars), stems (red bars) and leaves (green bars) of plants grown in NCS and CS at days 0 and 90. For each element, different lowercase letters in the bars within the same soil indicate significant differences in roots, stems and leaves, while different uppercase letters in roots, stems or leaves indicate significant differences among soils/days, both according to Duncan’s multiple range test (*p* ≤ 0.05).

The percentages of C, N, H and O in the different parts of the plants are illustrated in Figure 2. There were not available data of these elements for the leaves of plants grown in NCS after 90 days, this could be due to the difficulty for *J. curcas* L. (a plant from tropical origin) to bloom just after the cold winter of Seville.

**Figure 3:**
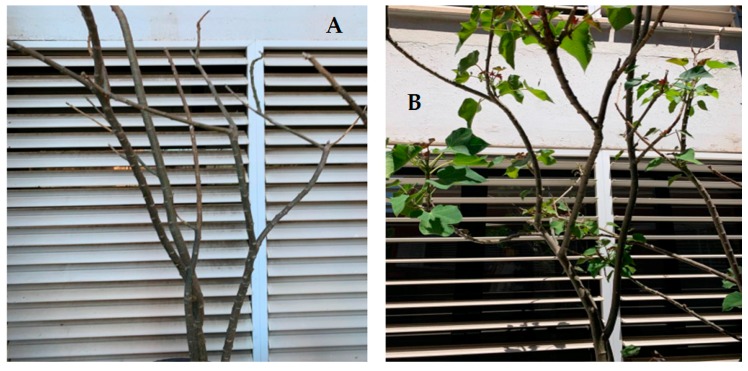
*J. curcas* L. in NCS (**A**) and in CS (**B**) after 90 days.

**Figure 4:**
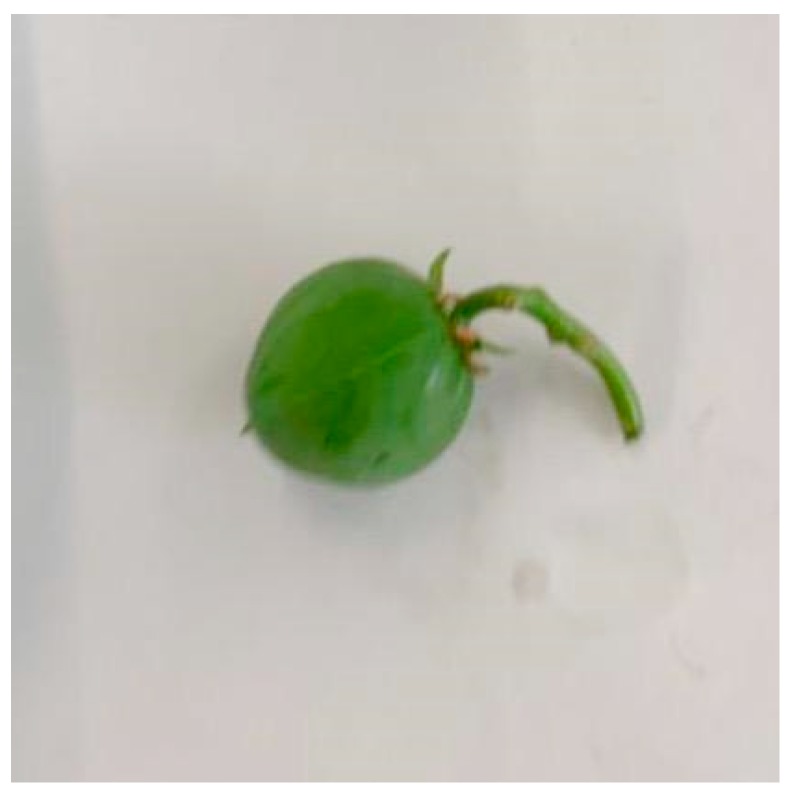
Seed from CS plant.

Of note is that the plants that had been planted in NCS did not have leaves on their branches after 90-day phytoremediation, while plants that has been planted in CS had leaves (Figure 3). Furthermore, seeds sprouted from plants grown in CS (Figure 4).

**Figure 5:**
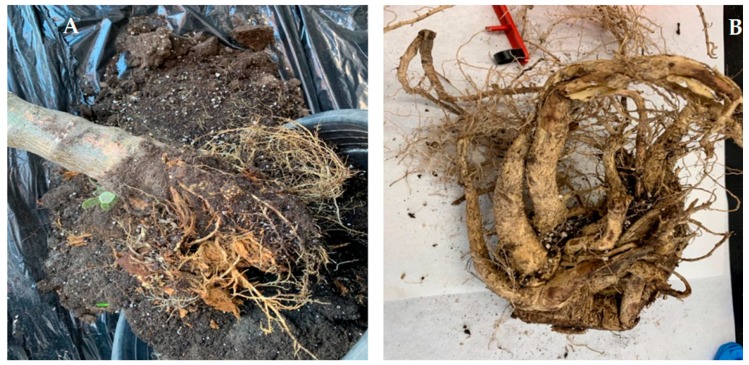
*J. curcas* L. roots from plants grown in NCS (**A**) and in CS (**B**) after 90 days.

On the other hand, the composition of the roots was also affected. Those from plants grown in CS had larger size and better structure, while roots from plants grown in NCS were smaller and weaker (Figure 5). It is worth noting that the plants used in this study were about four years old, so they had a very large stem size compared to the size of the roots.

**Figure 6:**
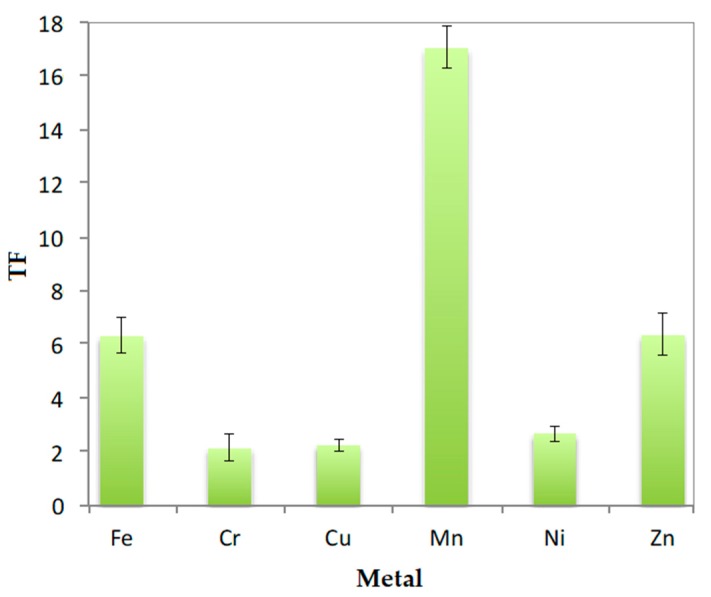
Translocation factors in the plant grown in CS after 90 days.

The translocation factors (TF) were calculated from the metal content in roots, stems and leaves. The TF obtained were higher than 1 for all the metals in *J. curcas* L. planted in both NCS and CS. The TF of the plants grown in CS after the 90-day phytoremediation period (data shown in Figure 6) were higher than those obtained in the NCS (data not shown), which can be justified by the presence of leaves in the plants grown in CS (higher aerial parts to root ratio).

**Figure 7:**
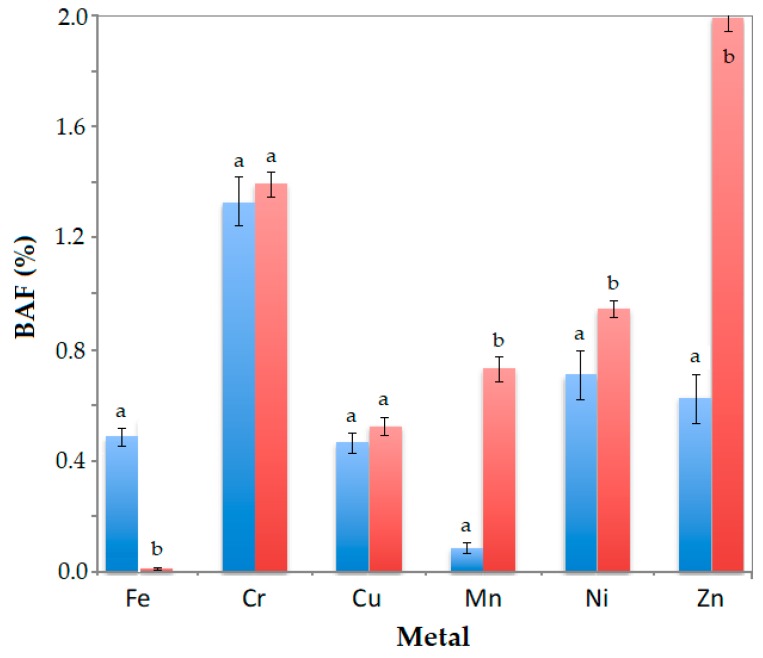
Bioaccumulation factors in roots (blue bars) and aerial parts (red bars) of *J. curcas* L. after 90 days planted in CS. Different letters in BAF bars of a metal indicate significant differences between BAF_root_ and BAF_aerial_ according to Duncan’s multiple range test (*p* ≤ 0.05).

Finally, from metals concentrations from both soils and plants, the bioaccumulation factors (BAF) were calculated. Independent BAF for roots (BAF_root_) and aerial parts (stem + leaves, BAF_aerial_) were established. Figure 7 shows the BAF for *J. curcas* L. planted in the contaminated soil.

## 3. Discussion

### 3.1. Evolution of pH in CS

The pH of CS started to increase from day 0 (Figure 1). This may be attributed to the ability of the plants to reach the soil balance. Therefore, if the plants were let grow longer time in the CS, this equilibrium would have been more noticeable, thus obtaining a final pH value very similar to that of NCS.

### 3.2. Metal Content Reduction in Soils

Similar reduction percentages of Fe, Ni and Zn were found in both soils (Table 2). Interestingly, the uptakes of Cr and Pb were higher for the plants grown in CS in spite of the fact that both CS and NCS had similar initial concentrations of these metals (Table 1), thus leading to higher percentages of reduction of Cr and Pb in CS (Table 2).

In a previous work on the phytoremediation by *J. curcas* L. of other highly contaminated soils, the plant reduced the Fe concentration of the different soil mixtures assayed by only 15–39% after 120 days, which was explained by the high initial Fe concentration (> 30000 mg kg^−1^) [11]. A similar Fe reduction in soils was achieved in this work, iron being the main metal found in the Santo António mining soil (Table 1). To be specific, *J. curcas* L. absorbed 38% of the Fe present in NCS, and 29% of the initial Fe in CS (Table 2). In the aforementioned publication the concentration of metals in soils with initial concentrations lower than 10 mg kg^−1^ decreased by 100%, while the reduction in concentrations of metals with initial concentrations between 10 and 1000 mg kg^−1^ ranged between 30 and 70% [11], with the exception of As, which agreed with the findings reported by other authors on the absorption of arsenic by *J. curcas* [12,13].

In the present work, a 44% As reduction was observed in the CS, contrasting to previous findings [11,12,13]. It is worth mention that the initial arsenic concentration in CS (4983 mg kg^−1^) was far higher than those of the soils used in the aforementioned references [11,12,13]. The As decrease in NCS was not available due to the very low initial As concentration in this soil (8 mg kg^−1^). With regards to Mn, Zn and Ni, their reductions were lower than the expected ones based on our previous work [11], especially for Ni, which concentration was expected to be almost nil after 90-day phytoremediation.

### 3.3. Elemental Analysis of Plants

Similarly to our previous work [11], the percentages of carbon and oxygen in the plants remained constant over time in NCS, with slight (but statistically significant) differences for CS. The same fact was expected for hydrogen. Nevertheless, a notable decrease of hydrogen in the leaves of contaminated plants was observed. Finally, when studying the nitrogen content, this decreased considerably after 90 days of treatment in plants planted in both soils.

The absorption of metals by adult *J. curcas* L plants affected mainly the nitrogen content, which increased in leaves and decreased in stems and roots (Figure 2). This is in agreement with the results found in the previous work on the *J. curcas* L. seedling and subsequent young baby plants growth in highly contaminated mining soils. In this case, and taking into account the data from Figure 2 and Table 3, an increase in nitrogen in leaves can also be observed (from 2.0% to 4.3%). This fact can be attributed to the chelating power of some metals, such as Cu or Zn, which can fix nitrogen to form complexes [14]. Notwithstanding, it can be observed that the amounts of the other three elements were similar. These results may be related to the physical changes visually observed in the plants (Figure 3, Figure 4 and Figure 5).

### 3.4. Translocation and Bioaccumulation Factors

The data obtained for plants grown in the metal-contaminated soil were considered for this discussion, since the growth and metal uptake of *J. curcas* L. in peat moss was not the aim of this research. High translocation factors were obtained in plants grown in CS (Figure 6). If the aim is to stabilize the metals in the roots so that they do not reach the stem or leaves (phytostabilization) and, therefore, prevent them from entering the ecosystem, TF much lesser than 1 should have been obtained. As can be seen, this fact did not occur for any of the analysed metals.

Considering TF values, it was expected BAF values would be higher in the aerial parts of the plant than in the root. According to Figure 7, it occurred for all metals except for iron, the main metal in the mining soil, which was more concentrated in the roots than in stems and leaves. Besides, it was expected that metals with higher TF values had a greater difference between BAF_aerial_ and BAF_root_. Except for Fe, this occurs for the analysed metals, mainly for manganese and zinc, the metals with the highest TF (17 and 6.3, respectively). Cr, Cu and Ni had lower TF (between 2.1 and 2.7). For them, the difference between BAF_aerial_ and BAF_root_ was lower (even without statistically significant differences at *p* ≤ 0.05 for Cr and Cu). Finally, it can be seen that chromium and zinc have BAF higher than 1. This may be related not only to the metal absorption from CS but also to the maturity of the plants. The plants were 4 years old before being planted in CS, so they absorbed a certain amount of these metals from the soil where they grew during 4 years and accumulated them in their tissues.

### 3.5. Potential of J. curcas L. for Phytoremediation.

From our previous work [11] and the current research, it can be stated that *J. curcas* L. can remove extremely high metal amounts from mining soils at any stage of its lifetime. Considering the TF and BAF achieved, *J. curcas* L. can be labelled as a hyperaccumulator plant. Hyperaccumulators absorb large amounts of heavy metals from the soil, which are not retained in the roots but are translocated to the shoots and accumulated in the aboveground organs at concentrations between 100 and 1000 folds higher than in non-hyperaccumulator plants [15]. However, as demonstrated in this and in our previous work [11], high metal concentrations, to certain extend, could not only not to pose toxic effects on plants, but also enhance their grow, based on the visual analysis (Figure 3, Figure 4 and Figure 5), which agrees with other authors’ findings [15,16]. Nevertheless, this metal accumulation in plant aerial parts makes *J. curcas* L. unsuitable for phytostabilization. *J. curcas* L. could be used for phytoextraction in mining areas, thus removing high amounts of heavy metals from these soils.

The main drawback is the later use of the metal-contaminated biomass generated over phytoremediation, i.e., it is mandatory to remove the contaminated plants from the soil. These contaminated plants are regarded as residues and are kept in vegetable containers, but a potential application for this biomass has been recently highlighted: the production of catalytic carbons. The pyrolysis of contaminated *J. curcas* L. roots under certain conditions leads to the production of biochar with graphite structure [11]. This biochar has been applied for the production of fuel oxygenated additives [17] and triglycerides [18] from residual glycerine from the biodiesel industry, and for the reduction of the acidity of oils containing high amounts of free fatty acids [19], the biocatalyst achieving similar performance to that of the commercial heterogeneous catalyst Amberlyst-15 for the same reactions. Therefore, and as conclusion, the phytoremediation of mining areas by *J. curcas* L. and subsequent production of biocatalysts could become an environmentally, economically feasible scheme.

## 4. Materials and Methods

### 4.1. Soil Samples

Soil samples were collected in Santo António mining area (Penedono, Portugal, 41°01′16.6”N, 7°24′27.4”W). The soils were screened in a 1 mm sieve thus eliminating all the stones present and homogenizing the sample. A mortar was used to reduce the size of soil particles and press them through the sieve. Next, a soil mixture was prepared by mixing 20% (wt.) mine soil with 80% (wt.) peat soil. The mixing of these soils was performed manually. This soil mixture was labelled as contaminated soil (CS).

### 4.2. Jatropha curcas L. Plants

Twelve plants from a previous research [11] were used in this work. After completing that research, the 12 plants were cultivated in peat moss in a garden, hence exposed to a direct Mediterranean climate without any controlled acclimatization, for 4 years before their use in this work.

### 4.3. Experimental Procedure

**Figure 8:**
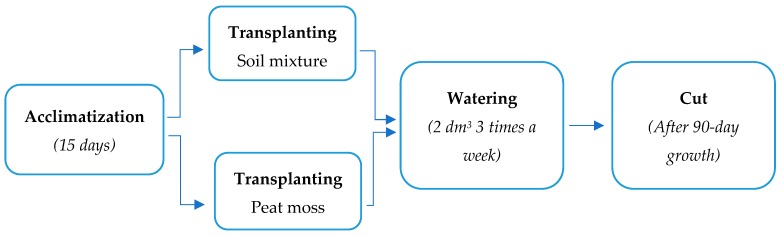
Experimental procedure.

First, the four-year-old *J. curcas* L. plants were planted in peat moss in 50-cm-pots and placed in the rooftop of the Faculty of Chemistry of the University of Seville on 8th March 2019 to achieve acclimatization to the environment for 15 days. Afterwards, eight plants were transplanted to their corresponding soils. To be specific, four plants were transplanted to 50-cm diameter pots with 6 kg CS, and the other four plants to 50-cm diameter pots containing 6 kg of non-contaminated soil (NCS), i.e., peat moss (Universal Compo Sana substrate, Münster, Germany). The pots were watered three times a week with 2 dm^3^ of tap water for 90 days. Four plants were cut-up and analysed at day 0. The other eight plants (four planted in NCS and another four planted in CS) were cut-up and analysed at day 90. Soils were analysed at days 0 and 90, i.e., 16 samples, four replicates of each soil at day 0 and another four replicates at day 90. The experimental procedure is summarized in Figure 8.

### 4.4. Plant and Soil Analysis

**Figure 9:**
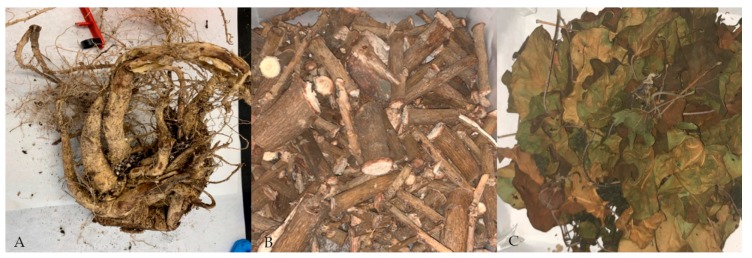
*J. curcas* L. (**A**) roots (**B**) stems and (**C**) leaves.

Prior to chemical analysis, plants were first cut-up; separating roots, stems and leaves (Figure 9). Subsequently, the three parts of the plants were dried in a stove at 60 °C to constant weight (approximately 72 h). Finally, they were ground using a Culatti DFH48 mill with a 1 mm sieve. As for soils, they were weighted and dried in a stove at 60 °C for 72 h. Once dried, the samples were sieved using a 1 mm mesh. A mortar was used to reduce the size of the soil particles and to press them through the sieve. After sieving, soil samples were homogenized.

#### 4.4.1. pH

In order to determine the pH of the soils, 10 g soil were weighed in a beaker and distilled water was added until obtaining a thick, homogeneous paste without excess water. This paste was then let stand for 30 min before introducing the pH meter electrode and acquiring the pH value.

#### 4.4.2. Metal Content

For the analysis of metals, both soil and plant samples were digested at 220 °C with concentrated HNO_3_ in an Ethos One microwave digester (Milestone Srl, Sorisole, Italy). After digestion, the amounts of Fe, Cr, Cu, Mn, Ni, Pb, Zn, As, Au and Sb were determined using a Spectroblue TI ICP-OES (Spectro Analytical Instruments GmbH, Kleve, Germany).

#### 4.4.3. Elemental Analysis

A TruSpec Micro elemental analyser (LECO Corporation, St Joseph, MI, USA) was used to determine the percentages of C, H, N, O and S in the plant samples. This method is based on the complete and instant oxidation of the sample by its combustion with pure oxygen at temperatures between 100 and 1000 °C.

### 4.5. Translocation (TF) and Bioaccumulation (BAF) Factors

The translocation of metals in plants was calculated as the ratio of the metal concentration in the aerial part of the plant (C_aerial_), i.e., stem, branches and leaves, to that of root (C_root_):

TF = C_aerial_ / C_root_ (1)

The plant is regarded to effectively translocate metals from root to the aerial parts for TF higher than 1. On the other hand, two bioaccumulation factors (BAF) were calculated. The BAF in root (BAF_root_) was defined as the ratio of metal concentration in the root (C_root_) to that in the soil (C_soil_), and the BAF in aerial part (BAF_aerial_) was calculated as the ratio of metal concentration in the aerial part of the plant (C_aerial_) to that in the soil (C_soil_):

BAF_root_ = C_root_ / C_soil_ (2)

BAF_aerial_ = C_aerial_ / C_soil_ (3)

### 4.6. Statistical Analysis

Data from the different Tables and Figures of the Results section were analysed by ANOVA. When ANOVA detected a significant (*p* ≤ 0.05) effect due to the factor studied, a 5% level of least significant difference, calculated by Duncan’s multiple range test, was used to establish differences between the mean values. Both ANOVA and Duncan’s multiple range tests were calculated by using Costat 2.10 software (Cohort Software, Berkeley, CA, USA).

## Figures and Tables

**Table 1:** Concentrations of metals (mg kg^−1^) and standard deviations (σ) in the starting soils.

Soil	Fe	Cr	Cu	Mn	Ni	Pb	Zn	As	Au	Sb
Mine	31985.1	1.29	39.8	35.9	ND	39.2	19.7	23716.7	2.12	2.76
σ	± 564.8^a^	± 0.34^a^	± 6.3^a^	± 4.0^a^	−	± 12.1^a^	± 3.2^a^	± 465.3^a^	± 0.23	± 0.46
NCS	7090.0	15.37	38.1	156.5	5.44	20.2	40.7	8.0	ND	ND
σ	± 204.5^b^	± 3.21^b^	± 3.0^a^	± 17.0^b^	± 0.98^a^	± 7.0^b^	± 16.0^b^	± 1.3^b^	−	−
CS	8331.2	13.41	38.7	59.6	1.14	22.6	41.5	4982.9	ND	ND
σ	± 265.3^c^	± 1.42^b^	± 4.8^a^	± 9.2^c^	± 0.36^b^	± 7.0^b^	± 15.9^b^	± 165.9^c^	−	−

ND: not detected. Different letters in the same column indicate significant differences according to Duncan’s multiple range test (*p* ≤ 0.05).

**Table 2:** Metal decrease percentages in NCS and CS soils after 90 days.

Metal	Concentration Decrease (%)	
	NCS	CS
Fe	38^a^	29^a^
Cr	54^a^	86^b^
Cu	43^a^	58^b^
Mn	45^a^	16^b^
Ni	28^a^	22^a^
Pb	42^a^	65^b^
Zn	28^a^	20^a^
As	ND^a^	44^b^
Au	ND	ND
Sb	ND	ND

ND: not detected. Different letters in the same row indicate significant differences according to Duncan’s multiple range test (*p* ≤ 0.05).

**Table 3:** Plant size at the beginning and at the end of the phytoremediation period.

Soil	Day	Height (cm)	Stem Diameter (cm)	Branch Diameter (cm)
NCS	0	221.8 ± 33.1^a^	27.5 ± 1.3^a^	17.3 ± 3.7^a^
NCS	90	225.8 ± 22.6^a^	26.8 ± 0.5^a^	13.0 ± 1.8^a^
CS	0	223.6 ± 25.1^a^	26.8 ± 1.7^a^	12.5 ± 1.3^a^
CS	90	220.5 ± 21.1^a^	26.8 ± 1.0^a^	13.8 ± 3.0^a^

Different letters in the same column indicate significant differences according to Duncan’s multiple range test (*p* ≤ 0.05).

## Abbreviations

BAF	Bioaccumulation factor
BAF_root_	Bioaccumulation factor in root
BAF_aerial_	Bioaccumulation factor in the aerial part of the plant
CS	Contaminated soil (peat and mining soil mixture)
ND	Not detected (under the detection limit)
NCS	Non-contaminated soil (peat moss)
TF	Translocation factor
